# Antimalarial and Cytotoxic Activity of Native Plants Used in Cabo Verde Traditional Medicine

**DOI:** 10.3390/plants12040963

**Published:** 2023-02-20

**Authors:** Anyse P. Essoh, Gustavo Capatti Cassiano, Filipa Mandim, Lillian Barros, Isildo Gomes, Márcia Melo Medeiros, Mónica Moura, Pedro Vitor Lemos Cravo, Maria M. Romeiras

**Affiliations:** 1 Linking Landscape, Environment, Agriculture and Food (LEAF) & Associated Laboratory TERRA, Instituto Superior de Agronomia, Universidade de Lisboa, Tapada da Ajuda, 1349-017 Lisboa, Portugal; 2 UNDP/UNFPA/UNICEF Joint Office of Cabo Verde-Energy, Environment and Climate Change Portfolio, Ed. Nações Unidas, Achada Santo António, Praia P.O. Box 62, Cape Verde; 3 Global Health and Tropical Medicine, Instituto de Higiene e Medicina Tropical, Universidade NOVA de Lisboa, 1349-008 Lisboa, Portugal; 4 Centro de Investigação de Montanha (CIMO), Instituto Politécnico de Bragança, Campus de Santa Apolónia, 5300-253 Bragança, Portugal; 5 Laboratório Associado para a Sustentabilidade e Tecnologia em Regiões de Montanha (SusTEC), Instituto Politécnico de Bragança, Campus de Santa Apolónia, 5300-253 Bragança, Portugal; 6 Instituto Nacional de Investigação e Desenvolvimento Agrário (INIDA), São Jorge dos Órgãos, Santiago CP 84, Cape Verde; 7 Research Centre in Biodiversity and Genetic Resources (CIBIO), InBIO Associate Laboratory, Pole of Azores, Faculdade de Ciências e Tecnologia, Universidade dos Açores, 9500-321 Ponta Delgada, Portugal; 8 Centre for Ecology, Evolution and Environmental Changes (cE3c), & CHANGE-Global Change and Sustainability Institute, Faculdade de Ciências, Universidade de Lisboa, 1749-016 Lisboa, Portugal

**Keywords:** tropical plants, traditional medicine, malaria, ethnopharmacology, West Africa

## Abstract

Medicinal plants have historically been a source of drugs in multiple applications, including the treatment of malaria infections. The Cabo Verde archipelago harbors a rich diversity of native plants, most of which are used for medicinal purposes. The present study investigated the in vitro antiplasmodial activities of four native plants from Cabo Verde (i.e., *Artemisia gorgonum*, *Lavandula rotundifolia*, *Sideroxylon marginatum*, and *Tamarix senegalensis*). Traditional preparations of these medicinal plants, namely aqueous extracts (infusions) and ethanolic extracts, were tested against both chloroquine-sensitive (3D7) and chloroquine-resistant (Dd2) *Plasmodium falciparum* strains using the SYBR Green detection method. The in vitro cytotoxicity was evaluated in Caco-2 and PLP2 cells using a sulforhodamine B colorimetric assay. An ethanolic extract of *A. gorgonum* and infusions of *T. senegalensis* exhibited high antiplasmodial activities (EC_50_ < 5 μg/mL) without cytotoxicity (GI_50_ > 400 μg/mL). Extracts of *L. rotundifolia* and *S. marginatum* exhibited moderate activities, with EC_50_ values ranging from 10–30 μg/mL. The *A. gorgonum* ethanolic extract showed activity toward early ring stages, and parasites treated with the *T. senegalensis* infusions progressed to the early trophozoite stage, although did not develop further to the late trophozoite or schizont stages. Antimalarial activities and the lack of cytotoxicity of the extracts are reported in the present study and support previous claims by traditional practitioners for the use of these plants against malaria while suggesting their ethnopharmacological usefulness as future antimalarials.

## 1. Introduction

Malaria infections are one of the major causes of death in the African continent [1]. *Plasmodium falciparum* is the most prevalent and lethal species that infects humans in the World Health Organization (WHO)–African Region, which possesses 29 countries where malaria is endemic, while it was responsible for 96% of the global malaria cases and deaths in 2020 [2].

The emergence of parasites resistant to currently used antimalarials [3], increasing resistance of vectors to insecticides, and the difficulty in developing efficient vaccines and therapies, underpin the urgent need for new means of malaria control. The current situation is especially alarming due to a convergence of threats, ranging from the COVID-19 and Ebola outbreaks to floods and other humanitarian emergencies, which have led to disruptions in national health service structures and impacted the treatment of several illnesses, including malaria [4]. Due to the fragility of health systems in several African countries and the limited access to physicians, the search for solutions in traditional medicine has increased considerably for diseases such as malaria [5].

Many plants have been used throughout the history of humankind as medicinal resources, as they constitute a rich reservoir of bioactive secondary metabolites that are still underexplored [6]. Nowadays, according to WHO reports, treatments with herbal medicine are still practiced by approximately 80% of the world’s population [7]. Although products derived from natural sources may not necessarily represent active ingredients in their final form, most commercialized drugs originated in nature [8,9]. For instance, the first treatment-based malaria control approaches came from traditional herbal medicine. Quinine, a component of the bark of the cinchona (quinaquina) tree, and its synthetic derivatives (chloroquine, hydroxychloroquine, amodiaquine, primaquine, and mefloquine) are some examples of the herbal medicines being used. Furthermore, artemisinin from the Chinese medicinal plant *Artemisia annua*, which is the predecessor of compounds such as artemether, arteether, sodium artesunate, and atovaquone, which is a synthetic analog compound (2-alkyl-3-hydroxynaphthoquinone) of lapachol from the *Tabebuia* species (Bignoniaceae) was also used [6].

Cabo Verde is a volcanic archipelago located in the Atlantic Ocean, with 10 islands located about 600 km off the West African coast (16°0′9″ N 24°0′50″ W). Cabo Verde was uninhabited until the 15th century and the geographical position of this archipelago, in the Atlantic Ocean, transformed these islands into an important port of call for the supply and repair of European vessels [10]. In the main port city of Santiago Island, Ribeira Grande, sailors from Portugal, traders on their way to Central America or Brazil, and travelers returning from the East, exchanged experiences and knowledge alongside some of the plants they had brought from distant locations, which served to prevent and cure most illnesses [10,11]. Several medicinal plants were introduced in Cabo Verde, mainly by immigrants of different ethnic groups from the West African regions [11]. The use of these plants to treat various diseases still constitutes an important health resource with significant potential for research and development of new medicines [12].

In Cabo Verde, malaria has been officially reported since the sixteenth century when the islands were settled [13]. As of January 2021, the country completed three consecutive years without local malaria transmission, becoming eligible to apply for the WHO certification of malaria elimination for the third time [2]. However, surveillance and prevention should remain in place because in Santiago Island, the most populated island, there is a constant flux of migrants from malaria hyperendemic regions, namely Senegal, São Tomé and Príncipe, Angola, and Guinea-Bissau [14].

Similar to other countries in Africa, traditional medicine has also played a role in fighting malaria in Cabo Verde, although to a lesser extent. According to De Pina et al. [14], only 3% of the population of the country has resorted to traditional remedies as a first-line treatment upon the appearance of the first malaria symptoms, although there is no reference to the number of people that carried on using traditional remedies while on conventional therapies, reflecting the strength of the public health system regarding malaria treatment.

In this study, four native species, *Artemisia gorgonum* Webb, *Lavandula rotundifolia* Benth., *Sideroxylon marginatum* (Decne. ex Webb) Cout., and *Tamarix senegalensis* DC., traditionally used as infusions for the treatment of malaria and malaria-like symptoms (e.g., fevers, shaking chills, and flu-like illness) in Cabo Verde, were investigated for their antiplasmodial activities, as well as for their cytotoxicity. Crude ethanolic and aqueous extracts (infusions) from these plants (i.e., leaves and stem) were tested in vitro against both chloroquine-sensitive (3D7) and chloroquine-resistant (Dd2) *Plasmodium falciparum* strains. Additionally, the in vitro cytotoxicity of these plants was evaluated in Caco-2 and PLP2 cells, using a sulforhodamine B colorimetric assay.

## 2. Results

The native medicinal plants chosen for this study were obtained from traditional healers and vendors of local markets of Santiago Island. The referred plants belong to different families and are used in treating different conditions such as fever, malaria, stomach aches, and infections.

### 2.1. Percentage Yield of Crude Plant Material

Table 1 summarizes the concentration and yield of eight extracts from the four medicinal plants tested in the present study. Each data represents an average of three replicates. *A. gorgonum* (20% for the H_2_O extract and 7.3% for the EtOH extract), had the highest extraction yield, both for infusions and ethanolic extracts, followed by *S. marginatum* (18% for the H_2_O extract and 5.7% the EtOH extract), and *L. rotundifolia* (18% for the H_2_O extract and 4.9% for the EtOH extract).

### 2.2. Antiplasmodial Activity

The antiplasmodial activity of the four medicinal plants commonly used in Cabo Verde was evaluated in vitro against two *P. falciparum* strains: the drug-sensitive 3D7 and the multidrug-resistant Dd2 (which are resistant to several antimalarials, including chloroquine, mefloquine, quinine, pyrimethamine, and sulfadoxine) and the results are shown in Table 2. Half maximal effective concentration (EC_50_) values ranged from 1.7 to ≥ 100 µg/mL.

Two of the plants exhibited high antiplasmodial activity (EC_50_ ≤ 5 µg/mL), namely the *A. gorgonum* ethanolic extract and the infusions of *T. senegalensis*. Ethanolic extracts of *T. senegalensis*, *L. rotundifolia*, and *S. marginatum* showed moderate antiplasmodial activities (EC_50_ > 5 to ≤30 μg/mL) against the 3D7 strain. Overall, ethanolic extracts were more potent than infusions, except for *T. senegalensis*. Interestingly, there were no significant differences in activity between the sensitive (3D7) and multidrug-resistant (Dd2) strains (*p*-value > 0.05), suggesting no cross-resistance between the extracts and antimalarials, such as chloroquine, pyrimethamine, and cycloguanil.

### 2.3. In Vitro Effect of A. gorgonum and T. senegalensis on P. falciparum Morphology

**Figure 1:**
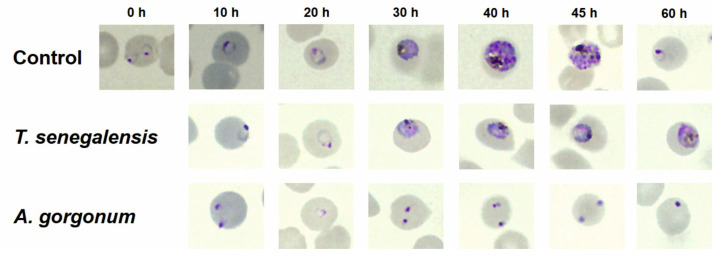
Treatment with infusions of *T. senegalensis* prevents trophozoite development. As can be seen, the parasite develops in the same way as the untreated control up to the 30 h time point. Treatment with *A. gorgonum* acts against early rings. In all time points, pyknotic nuclei were observed. Smears of synchronized 3D7 parasites treated with 5-fold EC_50_ concentration were stained with Giemsa. Untreated control is represented in the first row, where parasites developed according to the expected timeline.

To characterize the morphological changes in response to the two most active extracts (*A. gorgonum* ethanolic extract and infusions of *T. senegalensis*), synchronized *P. falciparum* 3D7 parasites were exposed to a concentration 5-fold greater than the respective extract EC_50_ value. Morphological changes could be observed 10 h after treatment indicating that the *A. gorgonum* ethanolic extract showed activity toward the early rings. No viable parasites were observed at the subsequent time points. Parasites treated at the ring stages with infusions of *T. senegalensis* progressed to the early trophozoite stage (20–30 h) but did not progress further to the late trophozoite or schizont stages (Figure 1).

### 2.4. In Vitro Hemolysis Assay and Cytotoxicity

**Figure 2:**
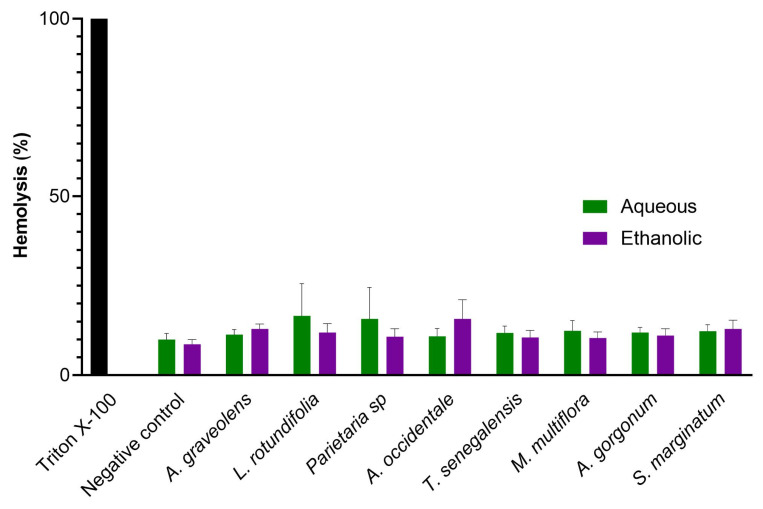
Hemolysis was measured in the presence of erythrocytes incubated with extracts at 50 µg/mL after 24 h. Triton X-100 was used a positive control. The data are derived from two independent experiments.

To confirm that the antiplasmodial activity of the extracts was not due to erythrocyte lysis, we performed a hemolysis assay. All plant extracts were non-hemolytic at the 50 µg/mL concentration (Figure 2). Furthermore, we evaluated the cytotoxicity of the two most potent plants against the parasite (A. gorgonum and T. senegalensis) using two cell lineages. Only the ethanolic extract of A. gorgonum showed moderate cytotoxicity against Caco-2 cells (GI_50_ = 17.3 µg/mL), while no toxicity toward the non-tumor PLP2 cells was observed (GI_50_ > 400 µg/mL) (Table 3).

## 3. Discussion

In the present study, we evaluated the antimalarial potential of medicinal remedies traditionally used for treating several diseases in Cabo Verde. Out of the four studied native plants (i.e., *Artemisia gorgonum*, *Lavandula rotundifolia*, and *Sideroxylon marginatum Tamarix senegalensis*), the extracts obtained from the leaves and stems of *A. gorgonum* and the leaves of *T. senegalensis* exhibited antiplasmodial activity without cytotoxicity, whereas *Lavandula rotundifolia* and *Sideroxylon marginatum*, exhibited only moderate activities.

It is important to stress that the studied species are among the most sold medicinal plants and are well recognized by the vendors at the local markets of the Santiago Islands for their therapeutic uses and health properties. The therapeutic administration recommended by the market vendors revealed that infusion (aqueous extraction) is the most common method of consuming these traditional remedies. However, there are some traditional practices that include the infusion of these medicinal herbs in “*grogu*” or “*grog*”, a highly alcoholic beverage of artisanal production [11]. We aimed at verifying the therapeutic potential of the extracts, comparing two different solvents at the same time of contact with the plant powder. Regarding the extraction yield, factors such as extraction method, the solvent used, contact time with the extraction solvent, and temperature, largely influence the extraction yield. Water, being the most polar solvent, was able to carry a higher quantity of phytochemicals than ethanol. According to several studies, solvent polarity significantly affected the extract yield, meaning that highly polar solvents produce a high extract yield when compared to less polar ones [15,16]. Water and ethanol are often recommended for extract preparation due to their differences in polarity and their lower probability to interfere with the phytochemical structure and properties [17]. Solvent polarity is also known to affect the quality of secondary metabolites and biological activities from the crude extracts, [18], a fact that was confirmed by a previous study using the same plant species [19].

**Figure 3:**
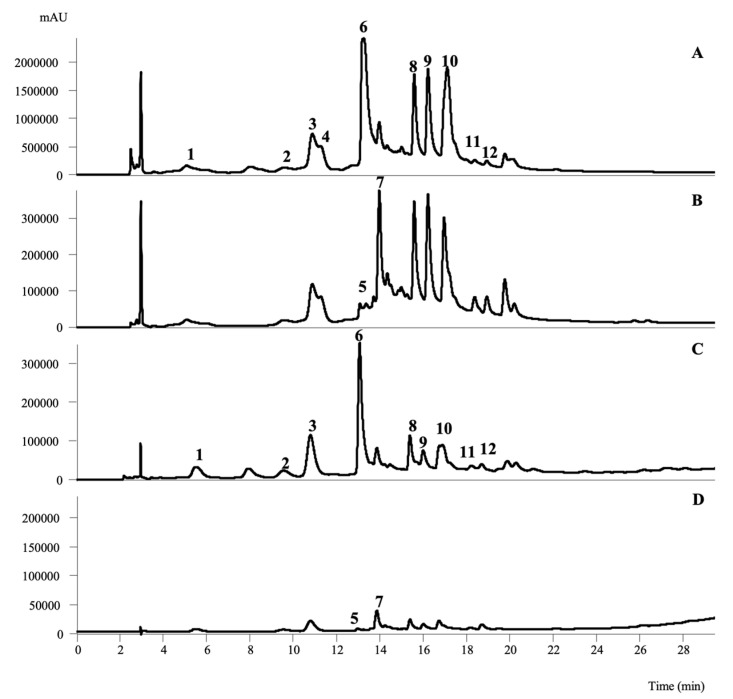
Phenolic profile of the infusion (**A**,**B**) and ethanolic (**C**,**D**) extracts of *Artemisia gorgonum* were recorded at 280 nm (**A**,**C**) and 370 nm (**B**,**D**), respectively. The identified peak number corresponds to the following compounds: **1**. 3-*O*-caffeoylquinic acid; **2**. 4-*O*-caffeoylquinic acid; **3**. *Cis* 5-*O*-caffeoylquinic acid; **4**. *Trans* 5-*O*-caffeoylquinic acid; **5**. Apigenin-6-C-Glc-4″-*O*-Glc (isosaponarin); **6**. Melilotoside; **7**. Apigenin-6-C-Ara-8-C-Glc (schftoside); **8**. 4-di-*O*-caffeoylquinic acid; **9**. 3,5-di-*O*-caffeoylquinic acid; **10**. 4,5-di-*O*-caffeoylquinic acid; **11**. *Cis* 3,4-di-*O*-caffeoylquinic acid; **12**. *Trans* 3,4-di-*O*-caffeoylquinic acid [19].

As shown in our previous study, elevated extraction efficiency was obtained for the infusions (aqueous extraction), presenting higher recovery of the phenolic compounds for all the studied species (between 9.5 and 100 mg/g extract) in comparison to the ethanolic extraction (between 6.90 and 13.3 mg/g extract) [19]. *A. gorgonum* exhibited a higher abundance and variety of phenolic compounds (100 mg/g extract), and of the twelve identified compounds, ten were phenolic acids and two were flavonoids. The 4,5-di-*O*-caffeoylquinic acid presented the highest concentrations in the infusions and melilotoside was the major molecule present in the ethanolic extract (Figure 3). Regarding *S. marginatum*, five phenolic compounds were tentatively identified, quercetin-*O*-hexosyl-deoxyhexosyl-pentoside was the most prevalent compound in both studied extracts (1.33 and 4.5 mg/g extract for ethanolic and infusions, respectively). Ferulic acid sulfate derivative was the molecule detected in the highest concentrations in infusions of *T. senegalensis* (10.7 mg/g extract), while for the ethanolic extract the flavonol, kaempferol-*O*-hexuronoside (1.59 mg/g extract), was the most abundant [19].

The results of the present study demonstrated that both the infusion and ethanolic extracts of some plants currently used in traditional Cabo Verde medicine exhibit in vitro antiplasmodial activity, with some of them being classified as highly active (EC_50_ ≤ 5 µg/mL) against *P. falciparum*, according to WHO standards and previous works [20,21]. The plant displaying the highest activity was *A. gorgonum*, namely in the leaves and stems. This species belongs to the list of Cabo Verde’s endemic plants, and although this is the first study testing its crude ethanolic extracts and infusions against malaria, its volatile oil had been tested previously, in vitro, for its antimalarial properties, having presented an EC_50_ of 5.2 µg/mL [22]. Later, 14 compounds were isolated from *A. gorgonum* leaves and stem and tested. Ridentin, sesamin, and artemetin were the most active compounds, showing an EC_50_ between 3.3 and 3.8 µg/mL against *P. falciparum* [22,23]. Some metabolites isolated from this species have also been tested regarding their anti-tumoral ability with promising results [24], as well as their hepatoprotective potential [25]. The *Artemisia* genus belongs to the Asteraceae family and 500 species have been reported, the majority of which are found in the temperate areas of Asia, Europe, and North America. Its use in traditional herbal medicine in several cultures is widespread, with many of its ethnomedical properties having been validated by conventional science. The Chinese *A. annua* was the species from which artemisinin, an endoperoxide sesquiterpene lactone, was first isolated, and at present, ACT (artemisinin-based combination therapy) is the first-line malaria treatment in most endemic countries. The identification of artemisinin represented a breakthrough in 20th-century tropical medicine, contributing to saving millions of lives in South China, Southeast Asia, Africa, and South America. The promising EC_50_ of the crude ethanolic extract of leaves and stems of *A. gorgonum* reinforces that this species can be a potential starting point for developing future antimalarial drugs.

The antiplasmodial activity of the plant extracts will sometimes depend on the concentration of some active antimalarial secondary metabolite(s). Gedunin, a highly active antiplasmodial metabolite (EC_50_ of 0.02 μg/mL) isolated from the leaves of *Azadirachta indica* A. Juss., is present at low concentrations in the plant, probably accounting for the moderate activity of its extract [26,27]. Alternatively, the ethanolic plant extract of *Artemisia afra* Jacq. ex Willd. displays an EC_50_ of 2.66 μg/mL [28], which is more potent than its isolated flavonoids acacetin, genkwanin, and 7-methoxyacacetin whose EC_50_ values ranged from 4.3 µg/mL to 12.6 µg/mL [29], suggesting that its secondary metabolites may act synergistically.

The present study also showed for the first time the antiplasmodial activity of *Tamarix senegalensis* infusion with EC_50_s of approximately 5 µg/mL against both sensitive and multidrug-resistant *P. falciparum* strains. Although several pharmacological activities have been demonstrated for other species of *Tamarix*, the limited use of this genus in traditional medicine may be due to the lack of clinical studies on its therapeutic and toxicological potential [30].

Overall, the antimalarial activities as well as the lack of toxicity of the extracts evaluated in the present study encourage further studies for the use of these plants against malaria and suggest their ethnopharmacological usefulness as future antimalarials. Nevertheless, sustainable management of Cabo Verde medicinal plants is important for their diversity conservation and to avoid their extinction, particularly in the case of highly used endemic endangered species (e.g., *Artemisia gorgonum*, *Lavandula rotundifolia* and *Sideroxylon marginatum* [31,32]) in traditional medicine.

## 4. Materials and Methods

### 4.1. Plant Material

All plants (Table 4) were purchased (in March 2021) at the two largest markets (i.e., Assomada’s and Praia markets) in Santiago, the largest and most populated island of the archipelago. These plants were identified and later housed in the LISC Herbarium (IICT/University of Lisbon) and transported to the Laboratory Ferreira Lapa (Institute of Agronomy) to prepare the extracts.

### 4.2. Plant Powder Preparation

To enhance the superficial contact and maximize compound content in the solvent media prior to the extraction, room-temperature dried plant samples (leaves and stems of *Artemisia gorgonum*, and leaves from *Lavandula rotundifolia*, *Sideroxylon marginatum*, and *Tamarix senegalensis*) were grounded in a lab-scale mixer (Bimby TM31, Vorwerk, Wuppertal, Germany) and manually sieved in the Analysette 3 Spartan apparatus (Fritsch, Idar-Oberstein, Germany).

### 4.3. Crude Ethanolic Extracts

The raw material (0.5 g) from each powdered plant was suspended in 10 mL EtOH (96%). The suspension was mixed thoroughly for 30 min at room temperature. After extraction, each solution was filtered under a vacuum using a qualitative paper filter (filter paper VWR 2–3 µm). The solvent was removed under reduced pressure using a rotary evaporator (BUCHI Rotavapor R-114, Lausanne, Switzerland) at 40 °C. The dried extracts were weighed and stored at 4 °C until further use.

### 4.4. Preparation of the Infusions

An aqueous extract was prepared by infusion, adding 10 mL of sterile distilled water at 95 °C to each powdered plant’s raw material (0.5 g) and left to rest for 30 min, manually shaking the mixture every 10 min and then cooling to room temperature. Each infusion was subsequently filtered using a Millipore 0.2 mm filter to remove particulate material. The resulting infusion was lyophilized in a Heto Dry Winner freeze dryer (Denmark).

### 4.5. Determination of Percentage Yield (%)

The percentage yield was determined by subtracting the weight of the flask containing the dry extract (W2) from the weight of the flask with dissolved extracts (W1), and dividing that value by the weight of the dried plant material (W0) using the following equation:

$$Yield\;\left(\%\right)=\frac{\left(W2-W1\right)}{W0}\;100$$

Extract/fraction was calculated as the average of three replicates.

### 4.6. Plasmodium Culture

Chloroquine-sensitive (3D7) and chloroquine-resistant (Dd2) strains were cultured in RPMI 1640 medium supplemented with 0.05 mg/mL gentamycin, 38.4 mM HEPES, 0.2% sodium bicarbonate, and 5% Albumax II, as previously described in a standardized protocol [38]. Then, erythrocytes were added to the culture to obtain a 5% hematocrit and incubated at 37 °C under a 5% CO_2_ atmosphere, with a daily exchange of medium. The parasitemia was monitored daily through microscopic observation of the Giemsa-stained smears. Synchronic cultures at the ring stage were obtained by two consecutive treatments at 48 h intervals with a 5% solution of D-sorbitol [39].

### 4.7. In Vitro Antiplasmodial Assay

Extracts were added in serial dilutions (100–0.05 μg/mL) into clear bottom 96-well plates (Costar #3904). *P. falciparum* cultures were added at a 2% hematocrit with 0.5% parasitemia and incubated for 72 h at 37 °C. Plates were frozen at −20 °C overnight. Lysis buffer (20 mM Tris-HCl, 5 mM EDTA, 0.008% saponin, 0.08% Triton X-100, and 0.4 μL/mL SYBR Green I dye (Invitrogen #S7585) was added to a new black 96-well plate. After the addition of 100 μL of lysate, the plates were incubated in the dark for 60 min and analyzed at 490 nm excitation and 540 nm emission wavelengths. EC_50_ was estimated by nonlinear regression analysis using Prism GraphPad software by plotting log dosing vs growth inhibition (expressed as a percentage relative to the drug-free control) and corresponds to the concentration of the extract responsible for 50% of antiplasmodial inhibition [40].

### 4.8. Morphological Changes in Parasites by Microscopy

In order to remove any left multinucleated schizonts, highly synchronous 3D7 parasites were obtained by Percoll treatment and subsequent sorbitol treatment (after 2 h of incubation). The remaining rings (0–2 h) were then maintained at 2% haematocrit and morphological changes of treated parasites were observed at different time points (0, 10, 20, 30, 40, 45, and 60 h).

### 4.9. In Vitro Cytotoxic Activity

The cytotoxicity of the extracts was assessed, as previously described by [41], using the sulforhodamine B (Sigma-Aldrich, St. Louis, MO, USA) colorimetric assay against Caco-2 cells (colorectal adenocarcinoma) (Leibniz-Institut DSMZ). A non-tumor primary culture obtained from pig liver (PLP2) was also tested. Ellipticine was used as a positive control. The results were expressed as GI_50_ values (μg/mL), which translates to the concentration of the extract responsible for 50% cell proliferation inhibition.

### 4.10. In Vitro Hemolysis Assay

In vitro hemolysis activity was evaluated as described by de Sena Pereira and colleagues [42]. Briefly, fresh RBCs were incubated with compounds and after 24 h the supernatants were collected, and the absorbance was measured at 540 nm. The hemolytic rate was determined in relation to the positive control (0.1% Triton-100), which caused 100% hemolysis.

### 4.11. Statistical Analysis

Experiments were performed in triplicate, and the results are expressed as mean ± standard deviation. These values were determined using Microsoft Excel. All statistical tests were performed using SPSS Statistics software (IBM SPSS Statistics for Windows, Version 22.0. Armonk, NY, USA: IBM Corp.), being analyzed using a Student’s *t*-test, with α = 0.05.

## 5. Conclusions

As malaria control efforts continue struggling with the evolution of parasites resistant to existing therapies, there is an urgent need for the search and development of new antimalarial compounds that may circumvent resistance, or that can be used in combination with traditional drugs. Out of the panoply of scientific strategic options for antimalarial drug discovery, plant secondary metabolites have traditionally yielded the most effective compounds, as exemplified by quinine and artemisinin derivatives. Therefore, there is a need to explore and advance work on plants that have already been used by local healers in endemic areas to treat malaria, and which may reveal interesting candidate bioactive agents that may ultimately serve as leads for the development of new effective antimalarials, following chemical optimization.

Traditional medicine plays an important role to treat a wide range of health problems in many countries in the West African region; nevertheless, little information is still available for many of them, above all in the case of Cabo Verde Islands. The present study, which focused on the largely unexplored phytochemical investigation of four native plants (i.e., *Artemisia gorgonum*, *Lavandula rotundifolia*, *Sideroxylon marginatum*, and *Tamarix senegalensis*) from Cabo Verde, as well as their respective ethnomedical validation, constitutes such an example strategy for the discovery of potential lead compounds for antimalarial drug development. Future studies on *Artemisia gorgonum* and *Tamarix senegalensis* will determine whether these plant species will be a promising source of antimalarial metabolites.

## Figures and Tables

**Table 1:** Percentage yield and concentration of infusions from the prepared ethanolic plant extracts.

Scientific Name (Plant Part Used)	Extraction Yield (%) H_2_O	Concentration (μg/mL) H_2_O	Extraction Yield (%) EtOH	Concentration (μg/mL) EtOH
*Artemisia gorgonum* (leaves and stem)	20	100.3	7.3	36.5
*Lavandula rotundifolia* (leaves)	18	90.1	4.9	24.5
*Sideroxylon marginatum* (leaves)	18	90.3	5.7	28.5
*Tamarix senegalensis* (leaves)	8	40.4	2.8	14.0

**Table 2:** EC_50_ values of studied plant extracts against *P. falciparum*.

Scientific Name	3D7		Dd2	
	EC_50_ (μg/mL) H_2_O	EC_50_ (μg/mL) EtOH	EC_50_ (μg/mL) H_2_O	EC_50_ (μg/mL) EtOH
*Artemisia gorgonum*	49.1 ± 12.3 *	1.7 ± 0.5	52.1 ± 11.3 *	3.3 ± 0.6
*Lavandula rotundifolia*	95.4 ± 13.2 *	26.3 ± 3.2	83.7 ± 19.6 *	32.0 ± 7.8
*Sideroxylon marginatum*	48.6 ± 5.6 *	25.6 ± 2.2	46.0 ± 8.7 *	32.0 ± 5.8
*Tamarix senegalensis*	4.7 ± 0.6 *	11.4 ± 2.1	5.4 ± 1.2 *	13.8 ± 3.3

* Statistical differences between aqueous and ethanolic extracts (*p* < 0.05, Student’s *t*-test).

**Table 3:** In vitro cytotoxicity activity of the ethanolic and aqueous extracts.

Cell Line	Solvent of Extraction	*Tamarix senegalensis* (GI_50_ (µg/mL))	SI	*Artemisia gorgonum* (GI_50_ (µg/mL))	SI
Caco-2	Infusions	>400	>85	181 ± 10 *	3.7
	Ethanolic	125 ± 4	11	17.3 ± 0.2	10
PLP2	Infusions	>400	>85	>400	>8
	Ethanolic	178 ± 3	15	>400	>235

Results are expressed as mean ± standard deviation. GI_50_ values correspond to the concentration of the extract needed to inhibit 50% cell proliferation. SI: selectivity index. Ellipticine (GI_50_ values in µg/mL): Caco-2: 1.21 ± 0.02; PLP2: 1.4 ± 0.1. * Statistical differences between aqueous and ethanolic extracts (*p* < 0.05, Student’s *t*-test).

**Table 4:** Plant species studied and their respective traditional uses *.

Species (Family)	Common Names	Native Status	Medicinal Applications	Parts Used	Preparation and Administration	Sample Voucher
*Artemisia gorgonum* Webb (Asteraceae)	Losna, absinto	Endemic	Intestinal & stomach problems, cough and respiratory diseases, fevers, malaria, diarrhea, and others	Leaves **, stems **	Infusion, vaporization, and smoked as a cigarette	Silva 2 (LISC Herbarium)
*Lavandula rotundifolia* Benth. (Lamiaceae)	Aipo, alfazema-brava, alpo-rotcha	Endemic	Pains, stomach and kidney problems, cough and respiratory diseases, fever, malaria, diarrhea, and others	Leaves *, flowers	Infusion, bath, and oral administration	Silva 9 (LISC Herbarium)
*Sideroxylon marginatum* (Decne. ex Webb) Cout. (Sapotaceae)	Marmulano	Endemic	Pains, fever and flu-like illness, rheumatism, bones	Leaves *, bark	Infusion, maceration, oral, and dermal administration	Silva 8 (LISC Herbarium)
*Tamarix senegalensis* DC. (Tamaricaceae)	Tarrafe, tamargueira	Native	Cough and respiratory diseases, fever, and flu-like illness	Leaves *, fruits	Infusion, bath, and oral administration	Silva 10 (LISC Herbarium)

* Bibliographic sources and online databases on medicinal applications of Cabo Verde’s studied species [12,19,31,33,34,35,36,37]. ** Plant parts used in the present study.

## Data Availability

We confirm that all data are original and provided in Tables and Figures within the article.
